# Dynamic Fluid in a Porous Transducer-Based Angular Accelerometer

**DOI:** 10.3390/s17020416

**Published:** 2017-02-21

**Authors:** Siyuan Cheng, Mengyin Fu, Meiling Wang, Li Ming, Huijin Fu, Tonglei Wang

**Affiliations:** 1 School of Automation, Beijing Institute of Technology, Beijing 100081, China; cheng901229@bit.edu.cn (S.C.); fumy@bit.edu.cn (M.F.); mingllove@bit.edu.cn (L.M.); 2120160891@bit.edu.cn (H.F.); Catkin72@bit.edu.cn (T.W.); 2 School of Automation, Nanjing University of Science and Technology, Nanjing 210094, China; 3 Beijing Automation Control Equipment Institute, Beijing 100074, China

**Keywords:** angular accelerometer, porous transducer, fluid transients, wave speed, dynamic permeability, streaming potential, sensor optimization

## Abstract

This paper presents a theoretical model of the dynamics of liquid flow in an angular accelerometer comprising a porous transducer in a circular tube of liquid. Wave speed and dynamic permeability of the transducer are considered to describe the relation between angular acceleration and the differential pressure on the transducer. The permeability and streaming potential coupling coefficient of the transducer are determined in the experiments, and special prototypes are utilized to validate the theoretical model in both the frequency and time domains. The model is applied to analyze the influence of structural parameters on the frequency response and the transient response of the fluidic system. It is shown that the radius of the circular tube and the wave speed affect the low frequency gain, as well as the bandwidth of the sensor. The hydrodynamic resistance of the transducer and the cross-section radius of the circular tube can be used to control the transient performance. The proposed model provides the basic techniques to achieve the optimization of the angular accelerometer together with the methodology to control the wave speed and the hydrodynamic resistance of the transducer.

## 1. Introduction

Angular acceleration plays a significant role in vibration detection, rotation controlling and navigation [1,2]. To achieve reliable and accurate direct angular acceleration measurements, different physical principles and technologies have been used: including superconductivity [3], floated-fly-wheel [4], MEMS [5], heat transfer [6,7], electromagnetics [8] and fluidics [9,10,11,12,13,14,15,16,17,18]. The fluid-based design demonstrates an excellent balance in accuracy, bandwidth, measurement range, volume and insensitivity to linear acceleration [9,10,11,12,13,14,15,16,17,18].

The fluidic channel is the fundamental structure in all fluid-based angular accelerometers, although different designs have been proposed [9,10,11,12,13,14,15,16,17,18]. The angular acceleration input is converted into differential pressure in the fluidic channel, which is measured precisely by a special transducer. In [9,10,11,12,13,14,15,16,17,18], different transducers have been carefully chosen to implement the pressure measurement. The molecular electronic transducer (MET) was utilized together with iodine-iodide electrolyte containing potassium iodide to transform the fluidic pressure into an electrical signal [9,10,11,12,13,14]. Wolfaardt [15] adopted a special spiral fluidic channel and a diaphragm transducer to measure angular acceleration. Our recent work reported a porous transducer of sintered glass microspheres to detect the differential pressure [16,17,18]. In sum, to design the fluidic channel and transducer to improve sensor performance in measurement range, linearity, accuracy and bandwidth is the key challenge, and sensor optimization requires current use of realistic theoretical models.

In the mathematical model of the sensor, the theoretical analysis of the generation and the propagation pressure in the fluidic channel, as well as the dynamic flow in the transducer is of great significance because they influence the sensitivity, measurement range and bandwidth. Huang et al. [9] regarded the fluid in an MET linear accelerometer as an integral part to deduce the relation between the acceleration input and the liquid flow. This assumption was also adopted and tested in our proposed liquid-circular angular accelerometer (LCAA) and was shown to be workable to derive the steady state pressure, but revealed limitations in the analysis of the dynamic properties of the fluidic system [16]. Wolfaardt [15] emphasized that compressibility and the pressure wave exert great effects on the dynamic flow in the fluidic system, further affecting the differential pressure on the transducer. He established a multi-degree of freedom (MDOF) model to describe the dynamic fluid in the channel and obtained several significant conclusions [15]. However, to guarantee the accuracy of the MDOF model, the degrees of freedom have to be large enough, which leads to excessive computation, and the MDOF model is more suitable to illustrate an angular accelerometer with a diaphragm transducer rather than a porous transducer. Recently, fluid flow in a circular tube has been modeled by our group on the basis of the theory of fluid transients for the first time, and a part of the primary conclusions has been obtained [17]. Regretfully, the model of the wave speed, the dynamic properties of the porous transducer and the transient response of the fluidic system were not considered, and these factors will be the main focus in this work.

This paper reports theoretical and experimental research on the fluid dynamics in the LCAA with a porous transducer. The structure, as well as the principle of the sensor is introduced first. In addition to the fluid transients in the circular tube [17,19,20], the model of the wave speed and the permeability of the transducer are discussed extensively. To verify the proposed model, prototypes are designed to implement experiments, and theoretical results are compared with the previous conclusions in [15,16]. Moreover, several crucial conclusions are obtained by applying the proposed model, which are beneficial to the performance improvement of the LCAA both in the frequency and time domains. Finally, some important indexes of the sensor performance are presented together with the calibration experiments.

## 2. Structure and Principle of LCAA

**Figure 1:**
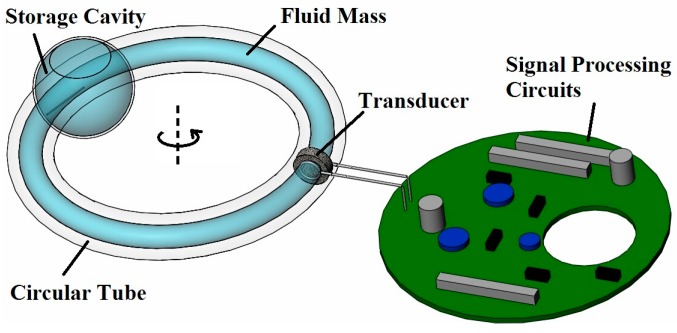
Structure of the liquid-circular angular accelerometer (LCAA).

The LCAA is designed as shown Figure 1. The circular tube is glass. The fluid is an organic liquid, and the porous transducer is sintered glass microspheres. When the circular tube rotates around the sensitive axis with angular acceleration, there is a relative motion between the fluid mass and the porous transducer, which consequently results in the generation of a differential pressure between the two sides of the transducer. The differential pressure forces the fluid mass to flow through the porous transducer, and the streaming potential emerges owing to the existence of the electrical double layer on the interface between the transducer and the fluid mass [21]. The organic liquid and the porous glass are adopted in LCAA because the combination of silicon dioxide and organic fluid has been discovered to generate easily measureable electrokinetic effect [22], and the porous material demonstrates satisfactory linearity between the differential pressure and the streaming potential [23,24]. A pair of metallic electrodes is mounted exactly close to the porous transducer, with a shape designed to avoid the resistance to the fluid flow. A storage cavity is provided, and a gas is provided to compensate the volume change of the fluid resulting from the temperature variation. Generally, the LCAA comprises a fluidic system and an electrical molecular system [16].

## 3. Theoretical Model of the Dynamic Fluid in LCAA

This section is concerned with the three parts of the theoretical model of the dynamic fluid in LCAA, including the fluid transients in the circular tube, the theoretical model of the wave speed and the dynamic permeability of the porous transducer. Methods to control the wave speed and the permeability are also explained.

### 3.1. Fluid Transients

Although fluid transients in the circular tube have been discussed in our previous work [17], the model is briefly illustrated here to keep the content of this paper easily understood and make the relation among the model of fluid transients, the wave speed and the permeability of transducer more distinct.

Fluid flow in the circular tube is assumed to be a one-dimensional and time dependent [19,20]. The *x*-axis is along the circular tube with x=0 at one surface of the porous transducer and x=2πR at the other side, where R denotes the radius of the circular tube. The transducer, whose thickness is neglected, is regarded as a porous jump. The density ρ and the velocity u of the fluid are time dependent. The equations of wave speed continuity and momentum are:

(1) $$a^{2}=\frac{K_{b}}{\rho }=\frac{\partial p}{\partial \rho }$$

(2) $$\frac{\partial \rho }{\partial t}+u\frac{\partial \rho }{\partial x}+\rho \frac{\partial u}{\partial x}=0$$

(3) $$\frac{\partial u}{\partial t}+u\frac{\partial u}{\partial x}+\frac{a^{2}}{\rho }\frac{\partial \rho }{\partial x}=0$$

where a is the wave speed in the fluid, $K_{b}$ is the bulk modulus and p=p(x,t) denotes the local pressure. The initial conditions are:

(4) $$\left\{ \begin{array}{l} u\left(x,0\right)=0\\ \rho \left(x,0\right)=\rho_{0} \end{array}\right.$$

where $\rho_{0}$ is the static density of the fluid. The volumetric fluid flow through the transducer is:

(5) $$q\left(t\right)=\left[v\left(t\right)-u\left(0,t\right)\right]\pi r^{2}$$

where r is the cross-section radius and v(t) is the transient velocity of the transducer derived from the angular acceleration input β(t):

(6) $$v\left(t\right)=R\int_{0}^{t}\beta \left(t\right)dt$$

Then, the definition of the hydrodynamic resistance of the transducer, $R_{h}$, is utilized to figure out the differential pressure on the transducer in Equation (7).

(7) $$\Delta p\left(t\right)=p\left(0,t\right)-p\left(2\pi R,t\right)=q\left(t\right)R_{h}$$

Under the assumption that the flow on the two sides of the transducer are equal, the boundary conditions could be obtained:

(8) $$u\left(0,t\right)=u\left(2\pi R,t\right)=v\left(t\right)-\frac{\Delta p\left(t\right)}{R_{h}\pi r^{2}}$$

### 3.2. Wave Speed

The wave speed in the circular tube exerts a vital influence on the dynamic performance of the LCAA since the natural frequency, $f_{n}$, of angular accelerometer is determined by the wave speed [15,17]:

(9) $$f_{n}=\frac{a}{4\pi R}$$

Thus, increasing the wave speed is beneficial to enlarging the bandwidth. The wave speed in a tube is affected by the material of the tube, the thickness of the wall and the gas in the tube [19,20],

(10) $$a=\sqrt{\frac{K_{eq}}{\rho \left[1+\left(K_{eq}D/Ee\right)\psi \right]}}$$

(11) $$\psi =\frac{2e}{D}\left(1+\upsilon \right)+\frac{D\left(1-\upsilon^{2}\right)}{D+e}$$

(12) $$K_{eq}=\frac{K_{liq}}{1+\left(V_{g}/V\right)\left(K_{liq}/K_{g}-1\right)}$$

where E is Young’s modulus of the material of the tube, Ψ is a non-dimensional parameter that depends on the elastic properties of the tube, υ is the Poisson ratio, D and e are the internal diameter and the wall thickness of the circular tube, respectively, $V_{g}$ is the volume of gas in the tube, V denotes the total volume of the fluid in the tube, $K_{liq}$ and $K_{g}$ are the bulk moduli of the liquid and the gas, respectively, and $K_{eq}$ is the equivalent bulk modulus of the fluid.

Additionally, the dispersion of the wave speed is known to occur when the tube wall is either rough or there is a porous medium in the tube [25]. In the situation where dispersion is relevant, the wave speed can be assumed to follow a normal distribution $a\sim N\left(\mu_{a},\sigma_{a}\right)$ rather than a single value. As can be seen from Equations (10)–(12), several parameters could be utilized to increase the wave speed, which include the ratio of the internal diameter to the wall thickness of the circular tube, the material of the liquid and the tube, as well as the volume of the gas in the circular tube. The influence of the structural parameters of the tube and the gas in the tube will be simulated in the experiments discussed below.

### 3.3. Dynamic Permeability Model of the Porous Transducer

Permeability is the most significant parameter of the porous transducer, since it affects fluid flow and the electrokinetic phenomenon [22,23,24,25,26,27]. The correlation between the hydrodynamic resistance of the porous transducer and permeability is defined by Darcy’s law [23],

(13) $$R_{h}=\frac{\Delta p}{q}=\frac{\eta H}{kA}$$

where η is the dynamic viscosity, H and *A* are the thickness and the cross-section area of the transducer, respectively, and k is the permeability. The hydrodynamic resistance of the transducer, $R_{h}$, is inversely proportion to the permeability and further affects the differential pressure and velocity (Equations (7) and (8)). The streaming potential coupling coefficient $C_{sp}$ (the ratio of the streaming potential, $E_{s}$, to the differential pressure, Δp), which could be used to serve as the model of the electrical molecular system, is influenced by permeability [26,27,28,29], and one model [29] gives:

(14) $$C_{sp}=\frac{E_{s}}{\Delta p}=\frac{\varepsilon_{0}\varepsilon_{r}\varsigma }{\eta \left(\sigma_{0}+\Sigma_{s}\sqrt{c}/\sqrt{kF}\right)}$$

where $\varepsilon_{0}$ and $\varepsilon_{r}$ are the permittivity of vacuum and the relative permittivity of the fluid respectively, ς is the zeta potential at the fluid-solid interface, $\sigma_{0}$ is the bulk conductivity of the fluid, $\Sigma_{s}$ denotes the surface conductance of the fluid-solid interface, c is a constant parameter determined by the pore shape and $F=\phi^{-m}$ is the formation factor of the porous medium, where ϕ is the bulk porosity and m is the cementation exponent of the porous transducer and assumed to be m=1.5 for spherical packing media [27]. As a result, the dynamic characteristics of the permeability can produce marked effects on the dynamic performance of the fluidic system, as well as the electrical molecular system of the sensor. Johnson et al. [30] has proposed a model of the dynamic permeability in porous material via analysis of permeability in the complex frequency domain, as:

(15) $$k\left(f\right)=\frac{k_{0}}{{\left\{1-\frac{4i\alpha_{\infty }^{2}k_{0}^{2}\rho f}{\eta \Lambda^{2}\varphi^{2}}\right\}}^{\frac{1}{2}}-\frac{i\alpha_{\infty }k_{0}\rho f}{\eta \varphi }}$$

where i is the imaginary unit, f is the operating frequency, $\alpha_{\infty }=F\phi$ is the tortuosity (defined to be the ratio of the actual length of the flow path in porous medium to the thickness of the medium along the pressure gradient) and Λ is a characteristic length scale in the transducer, which can be calculated from the average diameters of the microspheres, ${\bar{d}}_{p}$ [27],

(16) $$\Lambda =\frac{{\bar{d}}_{p}}{3\left(F-1\right)}$$

and $k_{0}$ is the permeability of the transducer in the steady flow determined by the Blake–Kozeny equation [24],

(17) $$k_{0}=\frac{\varphi^{3}{\bar{d}}_{p}^{2}}{150{\left(1-\varphi \right)}^{2}}$$

Combining the abovementioned models, the fluid dynamic model in LCAA is established. The model of fluid transients serves as the basic model when the wave speed in Equation (3) is calculated from Equations (10)–(12), and the hydrodynamic resistance of the transducer in Equation (8) is obtained from Equations (13)–(17). Analogous to [17], the method of characteristics can also be utilized with appropriate modification to solve these equations, which is not discussed in this work.

## 4. Experiments

In this section, we investigate experimentally several theoretical factors discussed above pertaining to wave speed and the entire system model for the transducer. The fabrication and experiments on the porous transducer are introduced first, followed by the simulation of the wave speed. Accordingly, the proposed model is applied to acquire the frequency response and transient response of the fluidic system, which are further verified experimentally in prototype systems. Moreover, the validity of the model makes it possible to analyze the influence of the structural parameters on the sensor.

### 4.1. Porous Transducer

#### 4.1.1. Fabrication and Permeability Measurement

Glass microspheres with three types of particle size distributions (PSD) were prepared first (Types 1–3), which are believed to follow the lognormal distribution [31,32]. Then, six types of microspheres (Types 4–9) were produced by selecting and mixing two types of microspheres in Types 1–3. The mixture proportions and parameters are shown in Table 1, where *d*(*α*) means that the total weight of the microspheres whose diameters are less than *d*(*α*) is *α* times the total weight of all microspheres. For example, *d*(0.5) = 34.39 μm denotes that the total weight of the microspheres whose diameters are less than 34.39 μm is 50% of the total weight of the all microspheres. The average diameter, ${\bar{d}}_{p}$, of every mixture type is also presented, which is defined by the Sauter mean diameter [33].

**Figure 2:**
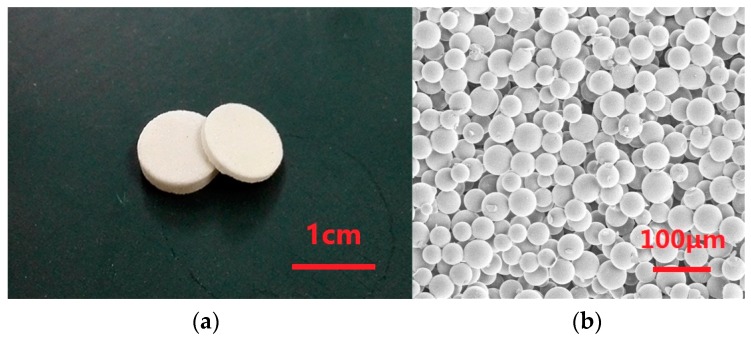
Transducers and the microstructure. (**a**) Appearance of the transducers; (**b**) microstructure of the transducer of Type 1.

All parameters of the microspheres were measured by a laser particle size analyzer (Mastersizer 2000), to specify the PSD since the basic PSD statistics, such as the origin moment and the Trask sorting coefficient [32], can be calculated from them. The porous transducers, depicted in Figure 2, were fabricated in molds by sintering the microspheres and mixtures in a furnace.

**Figure 3:**
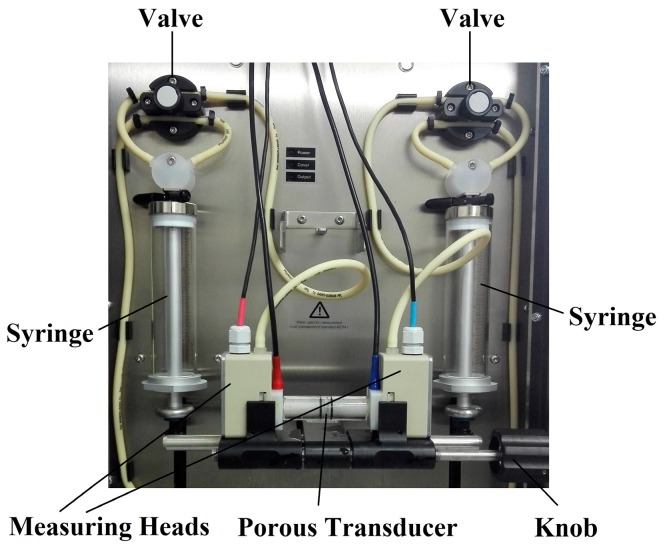
Instrumentation of the permeability measurement (SurPASS, Anton Parr Co., Graz, Austria).

Figure 3 shows the laboratory set up for the measurement of permeability. Transducers are mounted between the two measuring heads and placed in ring made of silica gel to avoid the wall effect [31]. The flow is controlled by the valves and syringes automatically, and the differential pressure on the transducer and the flow rate are measured at the same time to calculate the permeability by Equation (13). The measuring range of the differential pressure is ±100 kPa with the precision of ±(0.2% + 50 Pa). The flow rate can reach 300 mL/min.

**Figure 4:**
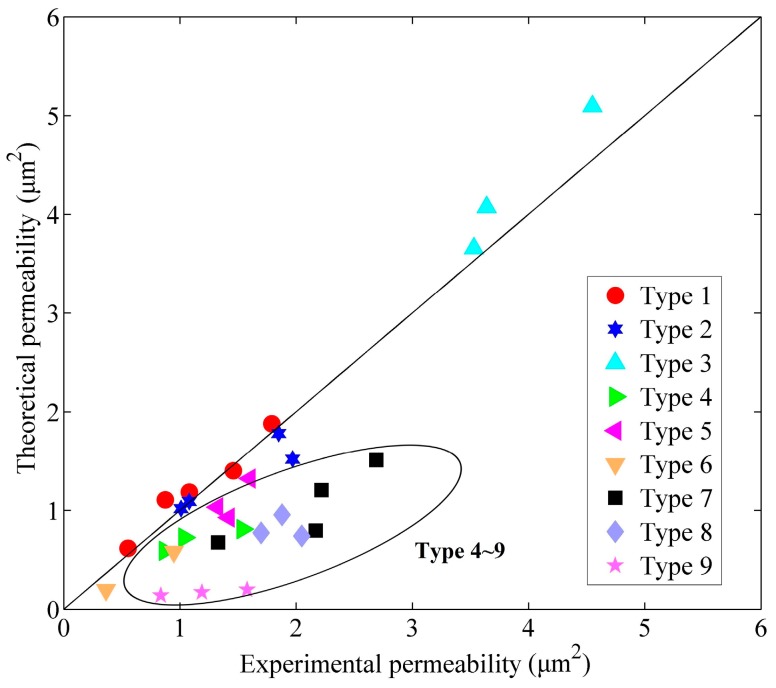
Experimental results of the permeability.

Each mark in Figure 4 compares the measured permeability of one transducer and the corresponding theoretical value evaluated by Equation (17). In this figure, the permeability of the transducers of the Types 1–3 coincides with the theoretical results, while the Blake–Kozeny equation gives an unsatisfactory prediction in Types 4–9. The mixture of glass microspheres of different PSDs probably results in unpredictable blocking in the micro channels in the transducer, which leads to the variation in the measured permeability. Furthermore, the transducers of Types 4–9 were more difficult to fabricate owing to dimensional deformation during manufacturing with an increased failure rate. Thus, transducers without a mixture of glass microspheres are the better choice for our LCAA due to their predictable permeability and superior structural quality. Hence, only the transducers of Types 1–3 were used in our experiments, and the permeability of the transducers used in this work is mainly 5 × 10^−13^ m^2^ < *k* < 9 × 10^−12^ m^2^.

#### 4.1.2. Experiments on the Streaming Potential

In addition to the permeability measurement, the SurPASS apparatus was also used to measure the streaming potential of the porous transducer. The measurement range is ±2000 mV with an accuracy of ±(0.2% + 250 μV). Although the magnitude of the streaming potential coupling coefficient, $C_{sp}$, shows differences due to the liquid selection, the non-dimensional results would still be analogous [25,26,27]. Therefore, a NaCl solution served as the test fluid in our experiment. For the reason that the streaming potential is influenced by the conductivity and pH of the liquid, the conductivity was held at 115 mS/m and pH = 6.2 in all of the experiments.

**Figure 5:**
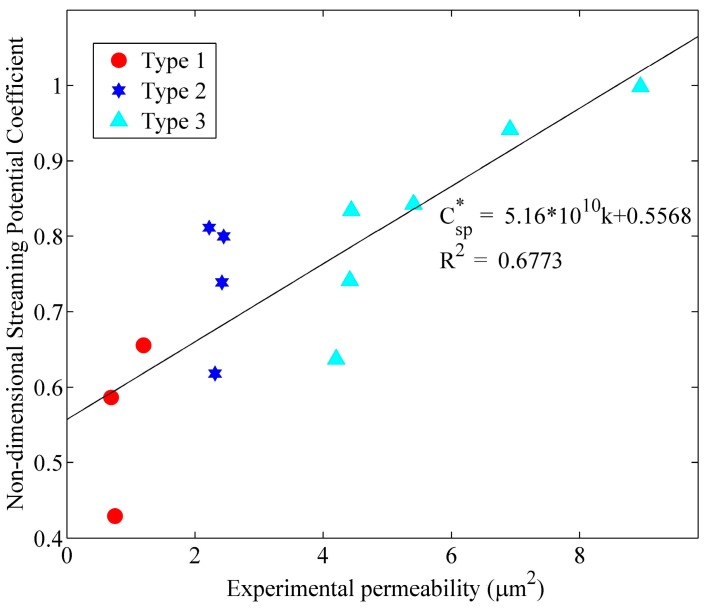
The relationship of streaming potential coupling coefficient versus experimental permeability.

In Figure 5, the streaming potential coupling coefficients of the transducers are normalized by the largest experimental value, $C_{sp}=-2.6\times 10^{-7}\;V/\mathrm{Pa}$. The normalized values, $C_{sp}^{*}$, are positively correlated with permeability. A similar correlation has also been noticed with other combinations of porous material and liquid [22]. Thus, increasing the permeability of the porous transducer contributes to the enlargement of the electrical signal on the electrodes.

#### 4.1.3. Simulation of the Dynamic Permeability Model

**Figure 6:**
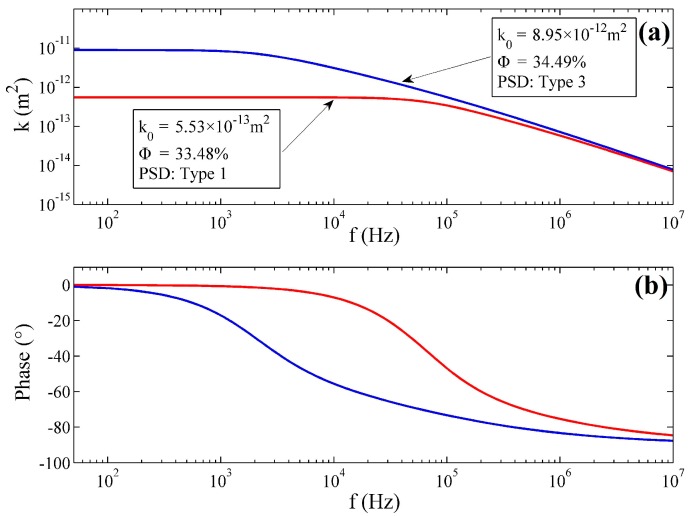
Frequency characteristic of the permeability. (**a**) Magnitude-frequency characteristic (MFC); (**b**) phase-frequency characteristic (PFC).

The dynamic permeability model of Equation (15) was next evaluated with the knowledge of the permeability range of the transducer. The simulation was conducted with the actual parameters of the transducers, which had either the largest or the least permeability. The permeability was analyzed in the complex frequency domain, and the frequency characteristics are displayed in Figure 6.

It can be concluded that the dynamic permeability model is similar to a first-order system that has a low-frequency gain and a corner frequency. The transducer with the larger permeability exhibits a smaller bandwidth since the corner frequency of the transducer with the largest permeability is near 1 kHz while the corner frequency of the other one approaches 100 kHz. Fortunately, the bandwidth of the transducer permeability is much larger than the bandwidth of the common fluid-based angular accelerometer (100 Hz) [9,11,14,15,16,17,18], as well as the best one (200 Hz) [34]. In most cases, it is not difficult to control the permeability of the transducer to maintain the bandwidth of the permeability apparently superior to the bandwidth of LCAA. Nevertheless, in some situations, if a porous transducer with large permeability or small hydrodynamic resistance is required to be applied in the sensor, the dynamic permeability would demonstrate great influence.

### 4.2. Simulation of the Wave Speed

**Figure 7:**
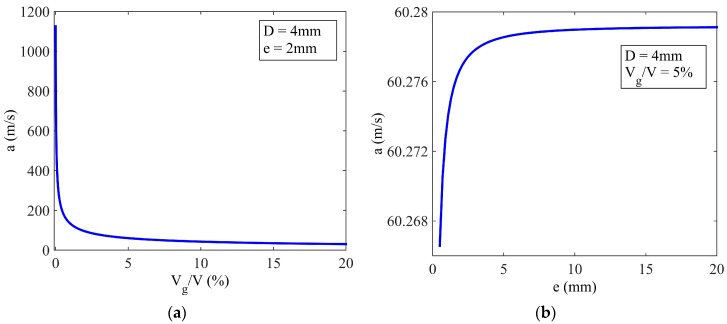
Wave speed in the circular tube. (**a**) The influence of the gas volume on the wave speed; (**b**) the influence of wall thickness on the wave speed.

The ideal wave speed is believed to be larger than 1000 m/s in water or some organic liquids [19,20]. However, the wave speed in the fluid-based angular accelerometer has been found much smaller than the ideal value, even less than 100 m/s [15,17]. The models in Section 3.2 were evaluated to analyze the impact of wave speed in the water flow in a glass circular tube. The relationships of the gas volume and tube wall thickness versus the wave speed are shown in Figure 7, and the parameters used in the simulation are listed in Table 2 [19,35]. It is evident that a little gas in the tube would lead to a great decay of the wave speed. When the gas volume is 5% of the total volume of the fluid, the wave speed in the liquid declines to 60 m/s. In Figure 7b, a thicker tube wall results in increased wave speed, although the influence is limited for *e* > 5 mm.

### 4.3. Simulation of the Frequency Response of the Fluidic System

**Figure 8:**
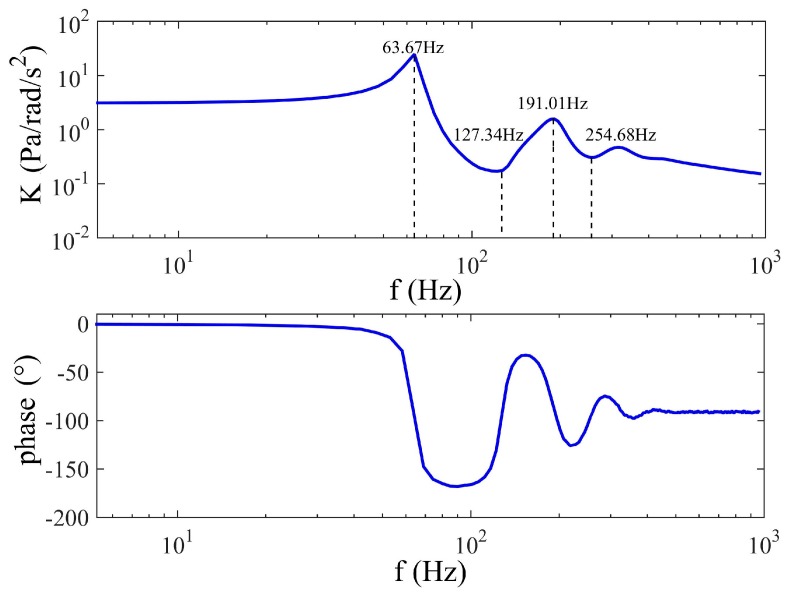
Theoretical frequency response of the fluidic system.

The proposed model, which contains the fluid transients, together with the theoretical and experimental conclusions about the porous transducer and the wave speed, is used to obtain a typical frequency response of the fluidic system for $R=25\;\mathrm{mm},D=8\;\mathrm{mm},\;k_{0}=1.27\times 10^{-12}\;m^{2}$, $H=2\;\mathrm{mm},\;A=25{\pi \;\mathrm{mm}}^{2}\;\mathrm{and}\;R_{h}=2\times 10^{10}\;\mathrm{Ns}/m^{5}$. The value of the wave speed does not influence the analysis because it mainly affects the natural frequency, which means a larger wave speed just makes a shift of the relation. To maintain consistency between simulation and experiment, the wave speed is set to be a=20 m/s to make the theoretical natural frequency close to the actual value of the LCAA or the prototype. Furthermore, the theoretical natural frequency can be known from Equation (9) as $f_{n}=63.67\;\mathrm{Hz},$ and the numerical result of the frequency response is plotted in Figure 8. In this figure, the first peak of the system gain is found to be located at the theoretical natural frequency, $f_{n}$, and there is a peak or a valley at each frequency that is the odd or even times $f_{n}$, respectively. In solid vibration systems [36,37], the *i*-th peak is called *i*-th order resonance, while the *j*-th valley is called *j*-th order anti-resonance, and they correspond to the different system modes. Considering the similarity seen here, the names of resonance and anti-resonance are also adopted to illustrate the frequency response of the fluidic system. The heights of the peaks in Figure 8 are found to be less than the heights in Wolfaardt’s work [15] because the dynamic permeability of the porous transducer is believed to bring non-negligible damping to the fluidic system. Thus, the quantity of the resonance is limited, which is different from that seen in [15]. Moreover, the phase of the system changes rapidly at every resonance of anti-resonance, although the phase beyond the fourth resonance tends to remain invariant.

### 4.4. Experiments of the Frequency Response of the Fluidic System

Prototypes were manufactured in ABS plastic to measure the output of the fluidic system, whose structure has been illustrated in [16,17], and additional prototypes have been produced in this work (Table 3). For the reason that this work focuses on the fluid dynamics in the circular tube and the transducer, the influence of the electrical molecular system is avoided. Therefore, the electrodes for streaming potential measurement are replaced by a pressure sensor that measures the differential pressure on the transducer. All of the transducers used in the prototypes have H=2 mm, $A=25{\pi \;\mathrm{mm}}^{2}$, and their permeability can be calculated from their hydrodynamic resistance. In these experiments, prototypes were produced to demonstrate the influence of R. For this purpose, the influence of the other parameters is suppressed; r is fixed; and the range of $R_{h}$ is maintained in a narrow range ($1.0\times 10^{10}\;\mathrm{Ns}/m^{5}-3.2\times 10^{10\;}\mathrm{Ns}/m^{5}$) to avoid unexpected effects of the different $R_{h}$ on the fluidic system.

**Figure 9:**
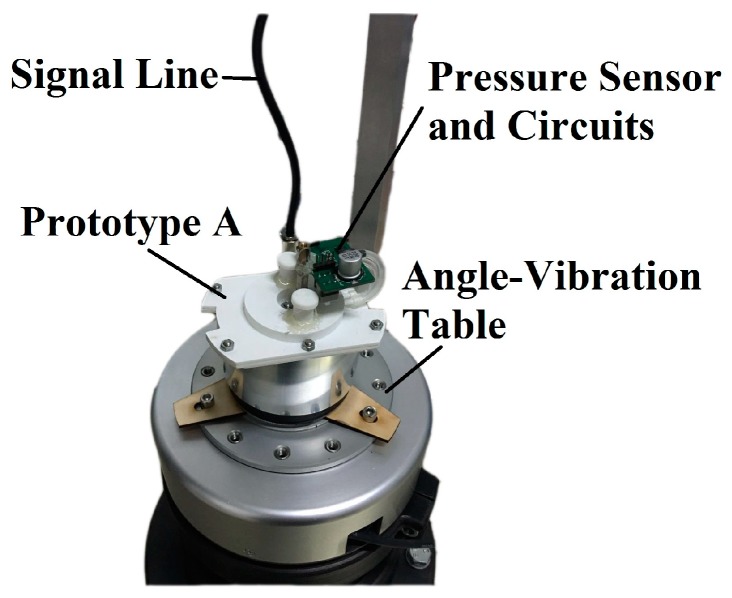
Experiments of Prototype A on the angle-vibration table.

The precise calibration of the angular accelerometer, which means that the relative error of the calibration process is lower than 0.5% and the bandwidth is larger than 500 Hz, is a challenging task in the world for several reasons. Firstly, it is not easy to define either the standard angular acceleration or to generate a constant angular acceleration. Secondly, the calibration requires the calibration platform to have enough bandwidth, as well as outstanding dynamic performance [2], which is affected by the error of measurement and control. Thirdly, it is difficult to determine the error of the calibration platform, and this error can only be estimated from the error of the measurement and control indirectly. Moreover, some researchers have presented the calibration process of their angular accelerometer, but did not discuss the performance of the platform [8,12,15], and some seemed to be bandwidth limited [12,15]. To date, the most accurate method is calibrating the sensor on an angle-vibration table equipped with either a grating scale or laser interferometer. Compared with the angle-vibration tables used in our previous work [16,17], a better one has been equipped in our laboratory recently, which is able to operate over 0.001–1000 Hz at several determined frequency points for both magnitude-frequency characteristic (MFC) and phase-frequency characteristic (PFC) measurements with relative accuracy of 0.5%. The calibration apparatus is shown in Figure 9. The prototype was fixed on the table, and the table generated sinusoidal angle vibration with accurate magnitude and frequency. The real-time output of the prototype was compared to the acceleration of the table, which was measured by the aforementioned optical method to obtain the gain and phase lag of the sensor. At every frequency, the prototype was tested with several angular acceleration inputs within the range 50–200 rad/s^2^ to examine the linearity of the gain, and it was found that the gain of the prototype with different angular acceleration inputs at the determined frequency maintained unchanged.

**Figure 10:**
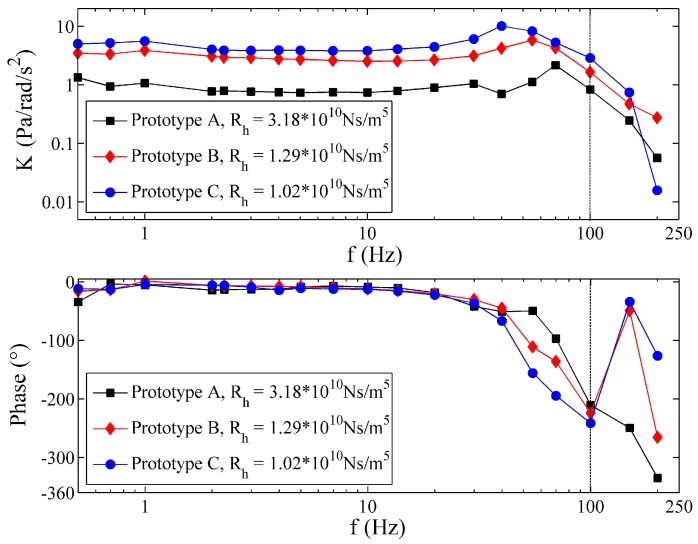
Experimental frequency response of the prototypes.

The frequency characteristics of the three prototypes are shown in Figure 10. It is not difficult to find the first resonance of every prototype in MFC and corresponding rapid changes near the natural frequency in PFC. The frequency characteristics of the fluidic system are thus apparently dependent on the radius of the tube, R. The prototype with the smaller radius exhibits a larger natural frequency, but smaller low-frequency gain, which is consistent with Equation (9) and the theory in [14,15,16,17]. However, when the operating frequency is larger than 100 Hz, the gain diminishes quickly, and the phase lag increases obviously. This unexpected phenomenon mainly results from the differential pressure measurement method in the prototypes, which transmit the pressure from the tube to the pressure sensor through holes and tubes. As a result, damping of the pressure magnitude and phase lag may be introduced into the whole prototype. For this reason, it becomes difficult to observe more resonances of the fluidic system, except the first one, although sometimes, they can be recognized in the experiments [17]. Fortunately, the performance of the fluidic system primarily depends on the first resonance, and the other resonances make a minor difference in the system.

**Figure 11:**
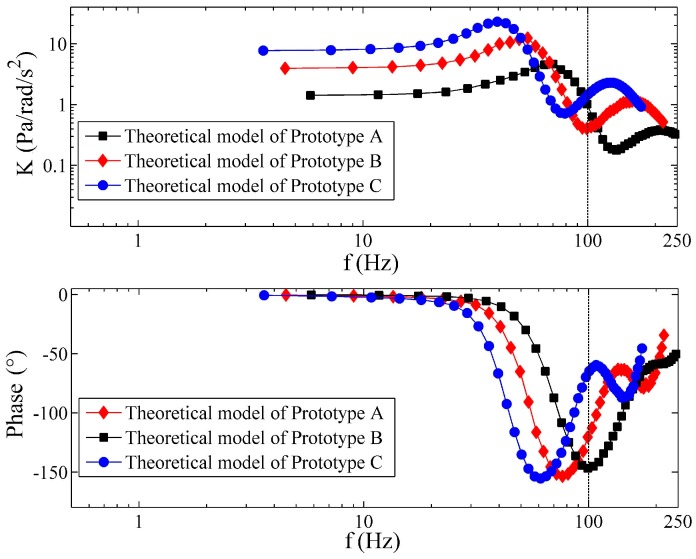
Theoretical frequency response of the prototypes.

The theoretical frequency responses of the three prototypes are shown in Figure 11. All of the structural parameters of the prototypes are utilized in the simulation to specify the theoretical model, and dispersion of the wave speed is included and assumed to follow a normal distribution as $a\sim N\;\left(\mu_{a}=15\;m/s,\sigma_{a}=10\;m/s\right)$ to get the best fit between the theory and experiment. Although the curves in Figure 11 are smoother than the measured curves, similar conclusions can also be obtained from Figure 11. Theoretically, the prototype with smaller R also performs with larger natural frequency, but smaller low-frequency gain. When the frequency is lower than the frequency of the first anti-resonance response in Figure 11, the changes of the PFCs coincide with the experimental results in Figure 10. The locations of the first resonance of the theoretical results are also consistent with those of the experimental data.

**Figure 12:**
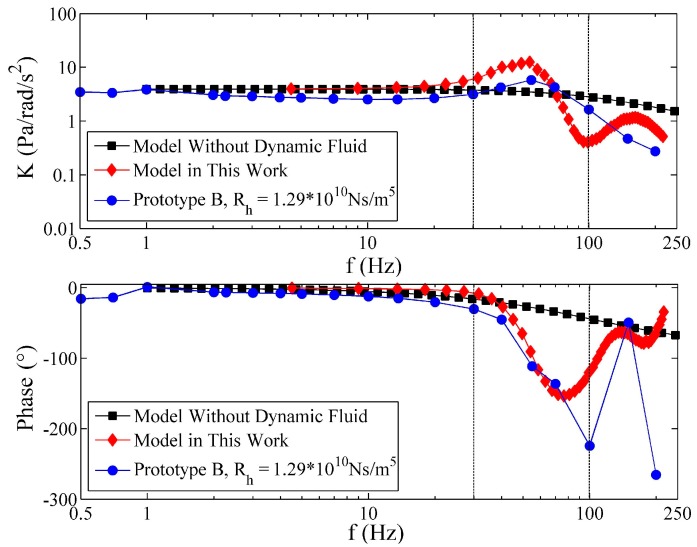
Comparisons among the experimental frequency response and the theoretical results of Prototype B.

Without loss of generality, the experimental frequency responses of Prototype B are selected for comparison with the proposed model of this paper and the previous model without fluid dynamic consideration [16]. In Figure 12, the two theoretical models show favorable consistency with the measurements in the low frequency range (*f* < 30 Hz). However, when *f* > 30 Hz, the model without dynamic fluid consideration is not convincing, because the location and the peak of resonance in MFC and the phase change in PFC cannot be predicted. On the contrary, the model in this work is almost able to predict the variation of the frequency response of the fluidic system when *f* < 100 Hz. As for the *f* > 100 Hz, the pressure loss and phase lag resulting from the pressure measurement may have an unexpected influence on the results, which is difficult to predict with the given measurement technique. Because of the pressure loss, the low-frequency gains of the experiments are slightly lower than the theoretical values. Hence, a loss factor is defined by the ratio of the theoretical low frequency gain, *K_th_*, of a prototype to the experimental low-frequency gain, *K_ex_*. The low frequency gain, *K_th_* = 2*πρR*^2^, is known from [16], and the same value of *K_th_* can be obtained by the proposed model in this work as shown in Figure 12. The average gain at the lowest three frequency points of each prototype is regarded as *K_ex_*. The results are shown in Table 4. The loss factor of each prototype varies in the range of 1.1–1.3, and the average loss factor is 1.2.

### 4.5. Experiment of the Transient Response of the Fluidic System

**Figure 13:**
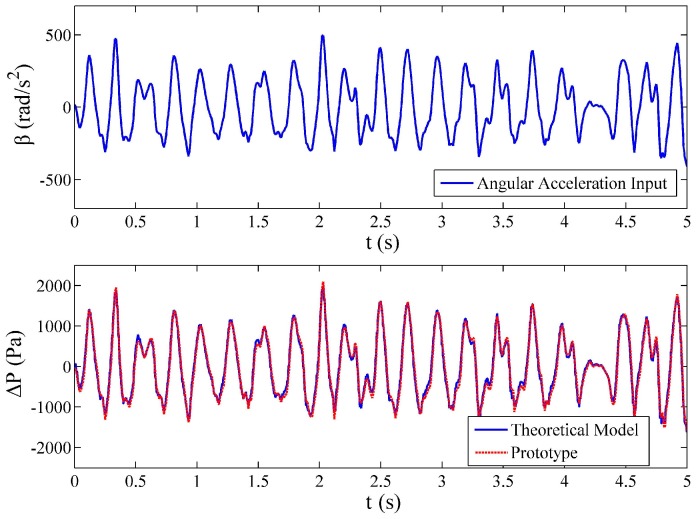
Comparison between the output of the prototype and the theoretical model responding to the same angular acceleration input. Prototype B, $R_{h}=1.29\times 10^{10}\;\mathrm{Ns}/m^{5}$, $V_{g}/V=5\%$, D=2r=8 mm, e=2 mm, $k_{0}=1.98\times 10^{-12}\;m^{2}$.

For the purpose of validating the output of the proposed model responding to the transient input, Prototype B was fixed to an arbitrarily rotating platform. The angular acceleration generated by this platform could be seen as a non-sinusoidal angular acceleration input to the prototype. The angular acceleration of the platform was detected by a fluid-based angular accelerometer, which is produced by the Beijing Automation Control Equipment Institute. It has good performance with a relative accuracy of 1%, a measurement range of −500~500 rad/s^2^, an output range is −12.5~12.5 V, a zero offset lower than 17°/s^2^ and bandwidth of 0.1–120 Hz. The signal detected by this accelerometer was regarded as the reference input signal and the output of the prototype was simultaneously recorded. To examine the transient performance of the proposed model, the reference input signal was inserted into the proposed model, in which the actual parameters of prototype were used with the ratio of the gas in the tube assumed to be 5%. Since the loss factor has been determined in Table 4, the differential pressure measured by the prototype was corrected by multiplying the average loss factor of 1.20. The transient theoretical output of the fluidic system is compared with the output of the prototype in Figure 13. It is noticed that the outputs of the theoretical model are consistent with the signal of the prototype with 1.4% relative error, and the details in the angular acceleration variation can be reflected by the theoretical model. In other words, the transient response of the fluidic system reacting to non-sinusoidal input can be predicted by the proposed model.

### 4.6. Influence of Structural Parameters

The proposed theoretical model of the fluidic system, which contains the fluid transients, the influence of the wave speed and the dynamic permeability of the transducer, has been shown to be valid. In the following, the theoretical model is applied to analyze the influence of structural parameters. Four indexes are considered to quantify the impact of the structure parameters on the fluidic system.

- Low frequency gain: the system gain at low frequency, which has an effect on the magnitude of the output signal of the system. Small low frequency gain would lead to the low signal-noise ratio of the sensor.
- Bode magnitude −3-dB bandwidth: the standard bandwidth of the system, which influences the operating frequency range of the sensor.
- Step response overshoot: relative height of the peak in step response, which mainly depends on the damping of the system.
- Step response transient time: transient time of the system changing into the 2% range of the new stable state in the step response.

#### 4.6.1. Influence of Hydrodynamic Resistance

**Figure 14:**
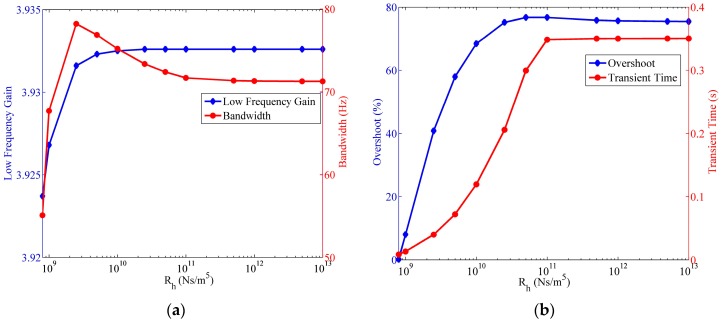
Relationship of the fluidic system indexes versus the hydrodynamic resistance of the porous transducer, where a=20 m/s, R=25 mm, r=4 mm. (**a**) Variation of the low frequency gain and the bandwidth; (**b**) variation of the step response overshoot and the transient time.

Figure 14 shows the influence of the hydrodynamic resistance on the four indexes. The size of the transducer is also set to be H=2 mm, $A=25{\pi \;\mathrm{mm}}^{2}$, and $R_{h}$ is determined when water is the fluid. The low frequency gain of the fluidic system is unchanged until the hydrodynamic resistance decreases to less than $10^{10}\;\mathrm{Ns}/m^{5}$. When compared with the slight variation of the low frequency gain, the bandwidth exhibits greater change when $R_{h}\leq 10^{11}\;\mathrm{Ns}/m^{5}$. The largest bandwidth is obtained with $R_{h}=2.5\times 10^{9}\;\mathrm{Ns}/m^{5}$, although the system bandwidth decays quickly with lower hydrodynamic resistance. Furthermore, the overshoot and the transient time of the step response demonstrates analogous correlation with $R_{h}$. If $R_{h}\leq 10^{11}\;\mathrm{Ns}/m^{5}$, less overshoot and transient time result from the reduced hydrodynamic resistance, which means that the fluidic system reacts to the step input more quickly.

**Figure 15:**
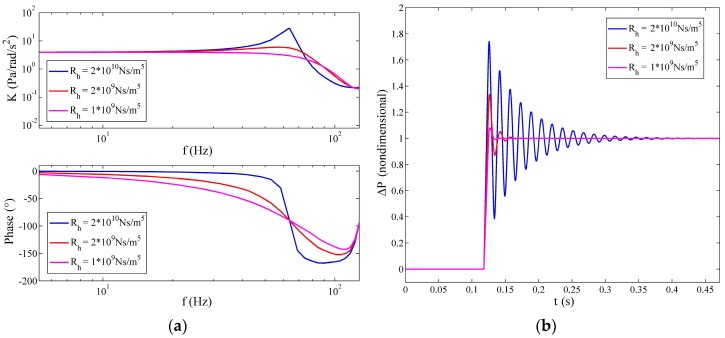
Changes of the frequency response and transient response resulting from $R_{h}$ variation, where a=20 m/s, R=25 mm, r=4 mm. (**a**) Variation of the frequency response; (**b**) variation of the step response.

The peak height of the system gain at the natural frequency is believed to impact the transient performance of the fluidic system. As shown in Figure 15a, with the reduction of $R_{h}$, the height of the first resonance of the fluidic system decreases and even disappears. Meanwhile, the fluidic system responds to the step input faster, and less oscillation emerges together with diminishing overshoot in the step response in Figure 15b. As a result, $R_{h}$ becomes a crucial parameter to optimize the dynamic performance of the fluidic system and the LCAA, and it is also convenient to control this parameter in the fabrication with the knowledge of Equations (13) and (17). Although it seems like it is a wise choice to increase the permeability to get a smaller $R_{h}$, as well as a better transient response of the fluidic system, the permeability is supposed to be controlled under 9 × 10^−12^ m^2^, since too large a permeability may result in the narrow bandwidth of the dynamic permeability, which has been explained in Section 4.1. Hence, the permeability, the hydrodynamic resistance and the size of the transducer should be designed together to achieve a good balance among the bandwidth of the permeability, the transient response performance, as well as the streaming potential coupling coefficient.

#### 4.6.2. Influence of the Wave Speed

**Figure 16:**
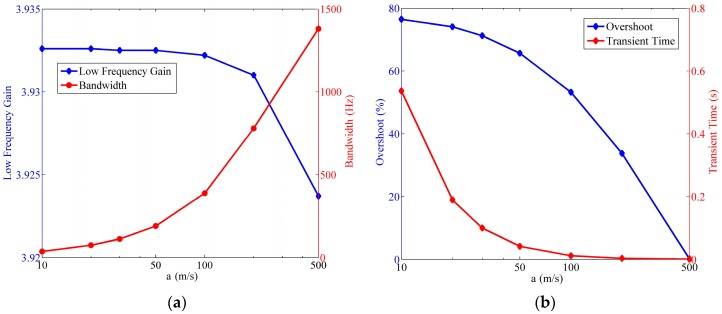
Relationship of the fluidic system indexes versus the wave speed, where $R_{h}=2\times 10^{10}\;\mathrm{Ns}/m^{5},$ R=25 mm, r=4 mm. (**a**) Variation of the low frequency gain and the bandwidth; (**b**) variation of the step response overshoot and the transient time.

As mentioned in previous sections, the wave speed imposes a prominent effect on the fluid flow in LCAA, and its influences are delineated in Figure 16.

The most evident conclusion is that despite the negligible deduction of low frequency gain, the increase of the wave speed contributes to the optimization in all of the other three indexes. It is the most efficient method to optimize the sensor by improving the wave speed, although great difficulties would be encountered within this process since both the roughness of the tube wall and the existence of the transducer limit the wave speed. As can be seen in Section 4.2, an effective technique to enlarge the wave speed at present is reducing the gas in the circular tube. Additionally, one thing should also be noticed, that the gas in the storage cavity might have a negative effect on the performance of the sensor. As a result, it is necessary to design a better structure in the future, which can not only deal with the volume variation of the liquid mass, but also avoid gas existence in the circular tube.

#### 4.6.3. Influence of the Radius of the Circular Tube

**Figure 17:**
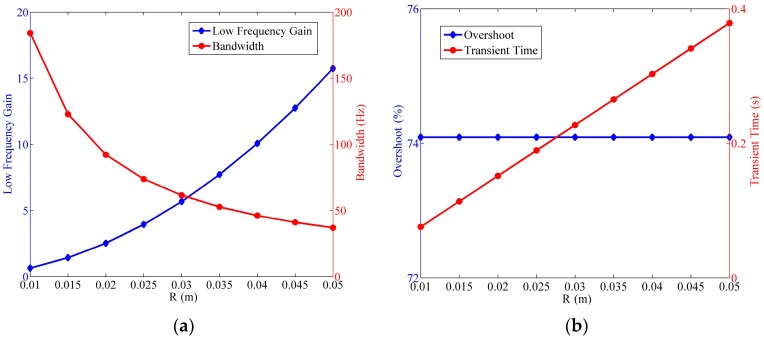
Relationship of the fluidic system indexes versus the radius of the circular tube, where $R_{h}=2\times 10^{10}\;\mathrm{Ns}/m^{5},$ a=20 m/s, r=4 mm. (**a**) Variation of the low frequency gain and the bandwidth; (**b**) variation of the step response overshoot and the transient time.

Since the natural frequency of the fluidic system relies on both the wave speed, as well as the radius of the circular tube, the influence of R is discussed in this section. In Figure 17, the low frequency gain and the bandwidth reveal contradiction with each other by changing R. Increasing the radius leads to the enlargement of the low frequency gain, but narrower bandwidth in the meantime. This phenomenon can also be found in the aforementioned results as Figure 10 and Figure 11. In engineering applications, a proper radius is supposed to be determined to obtain a good balance between the low frequency gain and the bandwidth. Besides, the overshoot is unrelated to R, while the transient time increases along with the radius.

#### 4.6.4. Influence of the Cross-Section Radius

**Figure 18:**
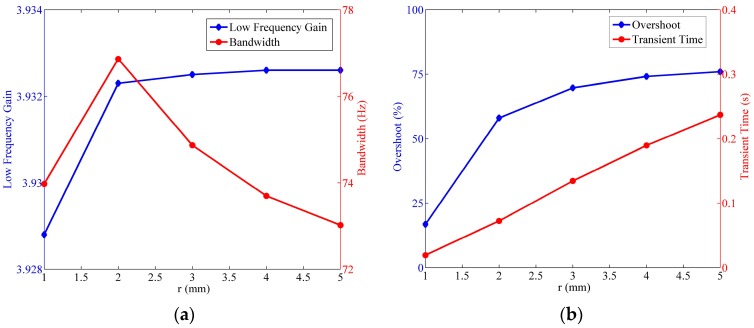
Relationship of the fluidic system indexes versus the cross-section radius of the circular tube, where $R_{h}=2\times 10^{10\;}\mathrm{Ns}/m^{5},$ a=20 m/s, R=25 mm. (**a**) Variation of the low frequency gain and the bandwidth; (**b**) variation of the step response overshoot and the transient time.

As can be known from the Equation (8), the impact of the cross-section radius of the circular tube r on the fluidic system is probably similar to the influence of $R_{h}$. The only difference is that the range of r is narrow due to the limit in the tube size. In the simulation, the largest bandwidth is found in Figure 18 when r=2 mm, and the low frequency gain is nearly invariant when r≥2 mm. Besides, the overshoot and the transient time manifest a positive correlation to the cross-section radius of the circular tube.

### 4.7. Performance of the LCAA

**Figure 19:**
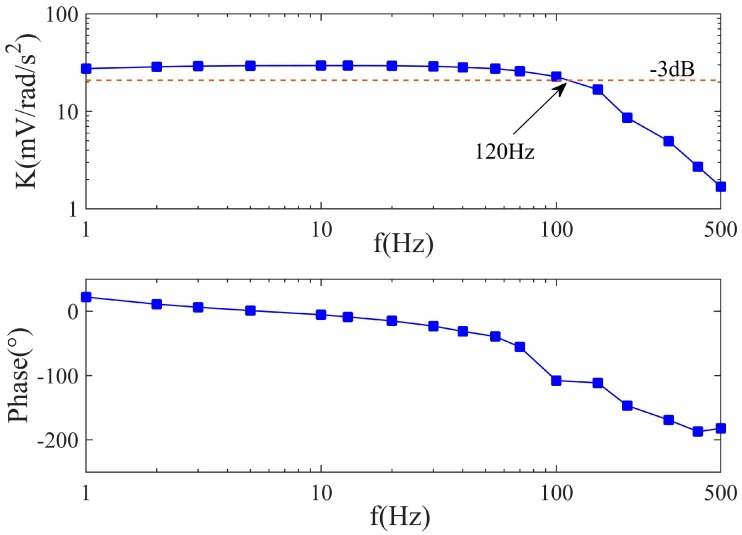
Frequency response of LCAA.

LCAA was also calibrated on the angle-vibration table, and this experiment was repeated and even conducted on some other calibration platforms to guarantee the reproducibility of the result. The frequency response of LCAA is drawn in Figure 19. As known from the discussion of Figure 15, the decreasing of the first resonance of the fluidic system is beneficial to the reduction of the overshoot and the transient time of the fluidic system, which can be controlled by the structure parameters of the sensor. Hence, with the knowledge in Section 4.6, appropriate structure parameters were selected to produce the sensor and the first resonance of the sensor disappears in Figure 19, which improves the transient performance of the sensor. Moreover, for the reason that the whole sensor is the combination of the fluidic system, the molecular electronic system, as well as the circuits, its frequency response differs from the fluidic system, but some features of the fluidic system could also be noticed if observed carefully. The first resonance always emerges before the first decay in MFC, which means the location of the natural frequency of the sensor lies in 70~100 Hz. A rapid change of PFC appears near the natural frequency and then becomes flat.

The fundamental indexes of LCAA are shown in Table 5. Compared with other types of angular accelerometer, this sensor performs with good balance in bandwidth, measure range, accuracy and environmental adaptation. According to the previous theoretical conclusions, it is also possible to enlarge the bandwidth by improving the wave speed or changing the radius of the circular tube, which would contribute to a more extensive application of LCAA.

## 5. Conclusions

The theoretical model of the dynamic fluid in LCAA has been presented in this paper, which mainly includes the model of fluid transients in the circular tube, the model of the wave speed and the dynamic permeability model of the porous transducer. This model can be applied to predict several characteristics of the fluidic system, such as the natural frequency, the locations of the resonances, as well as the anti-resonances, the low frequency gain and the transient response. On the basis of this mathematical model, the performance optimization techniques of LCAA could be further implemented. The gas in the tube, the wave speed and the radius of the circular tube can be better designed to control the low frequency gain and the bandwidth of the sensor while the hydrodynamic resistance, as well as the permeability of the porous transducer and the cross-section radius of the circular tube chiefly implement effects on the performance of the transient response.

The theoretical conclusions have been proven by the experiments, from which some other fundamental results have also been concluded. The microspheres of Types 1–3 are more suitable for transducer production, and the average diameter of microspheres could be used to identify the permeability of the transducer, which can further control the dynamic range of the permeability, the streaming potential and the hydrodynamic resistance of the transducer.

## Figures and Tables

**Table 1:** Parameters of the glass microspheres and mixture types.

Type	Mixture Proportion	*d*(0.1) (μm)	*d*(0.5) (μm)	*d*(0.9) (μm)	${\bar{d}}_{p}$ (μm)
1	-	23.94	34.39	49.53	33.08
2	-	36.99	52.62	74.69	50.68
3	-	56.99	80.15	112.32	77.35
4	Type 1:Type 2 = 1:1	30.83	45.61	67.44	43.57
5	Type 1:Type 2 = 1:3	34.92	50.14	71.78	48.19
6	Type 1:Type 2 = 3:1	25.59	38.68	59.03	36.85
7	Type 1:Type 3 = 1:1	33.11	57.91	99.38	52.82
8	Type 1:Type 3 = 1:3	36.15	71.31	123.20	60.43
9	Type 1:Type 3 = 3:1	22.56	40.50	79.64	37.21

**Table 2:** Parameters in the simulation of wave speed model.

Material	Young’s Modulus (GPa)	Poisson Ratio	Density (kg/m^3^)	Bulk Modulus (GPa)	Viscosity (mPa·s)
Glass	46	0.24	2500	-	-
ABS Plastic	1.7	0.33	1050 k	-	-
Water	-	-	998	2.19	1.01

**Table 3:** Parameters of different prototypes.

Prototype	*R* (mm)	*r* (mm)
A	15	4
B	25	4
C	35	4

**Table 4:** Determination of the loss factor.

Prototype	*K_th_* (Pa/rad/s^2^)	*K_ex_* (Pa/rad/s^2^)	Loss Factor (*K_th_*/*K_ex_*)
A	1.41	1.16	1.22
B	3.93	3.56	1.10
C	7.70	5.97	1.29
Average	-	-	1.20

**Table 5:** Indexes of LCAA.

Index	Value
Bandwidth	0.5~120 Hz
Measurement Range	−25,000°/s^2^~+25,000°/s^2^
Scale Factor	0.5 mVs^2^/°
Dead Band	1°/s^2^
Relative Error	1%
Power Supply	±15 V
Temperature Range	−40~+60 °C
External Size	Φ75 mm × 41 mm
